# Patterns of Protein Food Intake Are Associated with Nutrient Adequacy in the General French Adult Population

**DOI:** 10.3390/nu10020226

**Published:** 2018-02-17

**Authors:** Erwan de Gavelle, Jean-François Huneau, François Mariotti

**Affiliations:** UMR PNCA, AgroParisTech, INRA, Université Paris-Saclay, 75005, Paris, France; erwan.degavelle@agroparistech.fr (E.d.G.); jean_francois.huneau@agroparistech.fr (J.-F.H.)

**Keywords:** dietary protein pattern, nutrient adequacy, dietary diversity, protein diets

## Abstract

Protein food intake appears to partially structure dietary patterns, as most current emergent diets (e.g., vegetarian and flexitarian) can be described according to their levels of specific protein sources. However, few data are available on dietary protein patterns in the general population and their association with nutrient adequacy. Based on protein food intake data concerning 1678 adults from a representative French national dietary survey, and non-negative-matrix factorization followed by cluster analysis, we were able to identify distinctive dietary protein patterns and compare their nutrient adequacy (using PANDiet probabilistic scoring). The findings revealed eight patterns that clearly discriminate protein intakes and were characterized by the intakes of one or more specific protein foods: ‘Processed meat’, ‘Poultry’, ‘Pork’, ‘Traditional’, ‘Milk’, ‘Take-away’, ‘Beef’ and ‘Fish’. ‘Fish eaters’ and ‘Milk drinkers’ had the highest overall nutrient adequacy, whereas that of ‘Pork’ and ‘Take-away eaters’ was the lowest. Nutrient adequacy could often be accounted for by the characteristics of the food contributing to protein intake: ‘Meat eaters’ had high probability of adequacy for iron and zinc, for example. We concluded that protein patterns constitute strong elements in the background structure of the dietary intake and are associated with the nutrient profile that they convey.

## 1. Introduction

The importance of different protein sources in human nutrition has seen a renewal of interest because of sustainability issues that must be addressed in the near future [1]. In western countries, the subject has become highly topical since the development of various new dietary protein patterns in recent years, such as vegan, vegetarian and flexitarian diets [2,3]. Such patterns have been widely studied in specific populations for their association with the nutritional quality of diets [2,4,5] or mortality and morbidity [6,7,8]. However, very little is known about the profiles of protein intakes and the nutritional quality of diets in a more general, omnivorous population. 

The potential contribution of protein sources to nutrient adequacy is usually inferred from the nutrient profiles of different protein sources in terms of their differences and complementarity. As regards protein per se, animal foods are known to have higher protein:energy ratios and slightly better protein and amino acid digestibility than plant-based foods [9,10]. However, the differences between the nutrient profiles of protein sources are not limited to protein and amino acid intakes. Indeed, meat contributes more to zinc, vitamin B-12, phosphorus and iron intakes than plant-based products, whereas the latter are higher contributors of fiber, vitamin E or magnesium [11]. Accordingly, consuming a variety of protein food sources (meat, dairy products, fish, cereals, legumes) is advocated to ensure adequate nutrient intakes, as recommended by national guidelines [12,13].

However, protein sources cannot be studied as if they are simply added together, because they form part of complex diets. The intake of each protein food group is associated with that of other food groups, but this complexity can be described using the dietary patterns approach. Dietary patterns based on the overall food intake have been widely studied during the past decade relative to health outcomes or the quality or diversity of diets [14,15]. These studies have often identified three to six dietary patterns from population food intakes, such as the Mediterranean, ’Prudent’, ‘Nordic’ or ‘Western’ patterns [14,16]. In most cases, these patterns are identified using individual overall food group intakes in order to describe and summarize the highest number of food factors. 

However, few studies have adopted the same approach to analyze protein food intakes and reduce the multidimensionality of protein intake to a limited number of protein food profiles [17,18]. These studies identified five to six clusters based on their protein profiles and assessed the link between these profiles and health parameters, but they did not study associations with the nutritional quality of different diets. 

Our aim was to identify and analyze food protein profiles in a general western population and study their associations with the nutrient adequacy and diversity of the diet. 

## 2. Materials and Methods 

### 2.1. Population and Food Data

The population studied was derived from that covered by the second individual and national food consumption survey (INCA2) performed in 2006–2007, as previously described [19]. Briefly, we excluded adults over 65 years old (whose nutrient requirements differed from those of younger adults) and under- and over-reporters, which led to a final sample containing 1678 adults (717 men and 961 women). Food intakes were derived from 7-day food records, and individual characteristics from self-reported and face-to-face questionnaires, as described by Dubuisson et al. [20].

### 2.2. Nutrient Composition of Foods

Data on the nutrient composition of the INCA2 foods were extracted from the 2016 CIQUAL (Centre d’Information sur la Qualité des Aliments—Centre for Information on Food Quality) database, with the following modifications. Niacin equivalent was calculated as the sum of preformed niacin and 1/60 tryptophan, which was extracted from the amino acid database described in detail elsewhere [19]. The phytate composition was derived from and adapted to French foods from the phytate content of British foods table developed by Amirabdollahian et al. [21]. Percentages of heme and non-heme iron in animal foods were obtained using reports from the French Information Centre on Meat [22,23] and other published analyses [24]. The estimated bioavailability of protein, zinc and iron was taken into account given the differences between animal and plant sources, as described previously for protein [19], iron [25,26] and zinc [27]. The complete method implement is described in Methods S1.

### 2.3. Identification of Dietary Patterns

Dietary patterns were identified based on the intakes of “protein” food groups. INCA2 food items were classified as “protein foods” if they met two criteria: (1) the percentage energy from protein was >10%, which refers to their intrinsic protein content, (2) the level of intake at the 90th percentile was >5 g protein, which refers to their potential contribution to protein intake at a relatively high level. 

“Protein” food groups were selected from the 123 INCA2 food sub-groups which complied with two criteria: (1) >25% of the food items in the sub-group were “protein” food items as previously defined, and (2) the food groups were consumed by >10% of the population, in order to avoid an excessive number of “zeros” in the data which might lead to irrelevant results, and as applied during previous studies [16,28]. The groups were redefined to aggregate certain foods with a similar composition and usual time of consumption (e.g., “ripened cheese” and “non-ripened cheese” into “cheese”), or extract food sub-groups described in the recently updated French National Nutrition and Health Program (PNNS) nomenclature [12] (e.g., “lean fish” and “fatty fish” from “fish”). Forty-three protein groups were finally selected to identify dietary protein patterns (Table 1).

The non-negative matrix factorization (NMF) method was applied to the percentage contribution of each sub-group to protein intake. The NMF method was designed to summarize information on the 43 protein sub-groups in a limited number of factors representing combinations of protein subgroups eaten by similar individuals. We chose to use the term “factor” rather than “consumption systems” which had previously been applied in several studies with a similar NMF method [16,29,30] as the term “consumption system” could be understood as being related to food origin, cooking method or consumption location. The “loadings” of the protein food subgroups on the factors were the weight, or the contribution, of each protein subgroup on the factor. The Brunet algorithm [31] was implemented, choosing the number of factors from graphical analyses [16,29] and taking account of the interpretability of the results. Finally, a hierarchical cluster analysis was performed on the score achieved for the eight factors by each individual. The number of clusters chosen was based on both the elbow and silhouette [32] methods.

### 2.4. Nutrient Adequacy of the Diet

**Figure 1:**
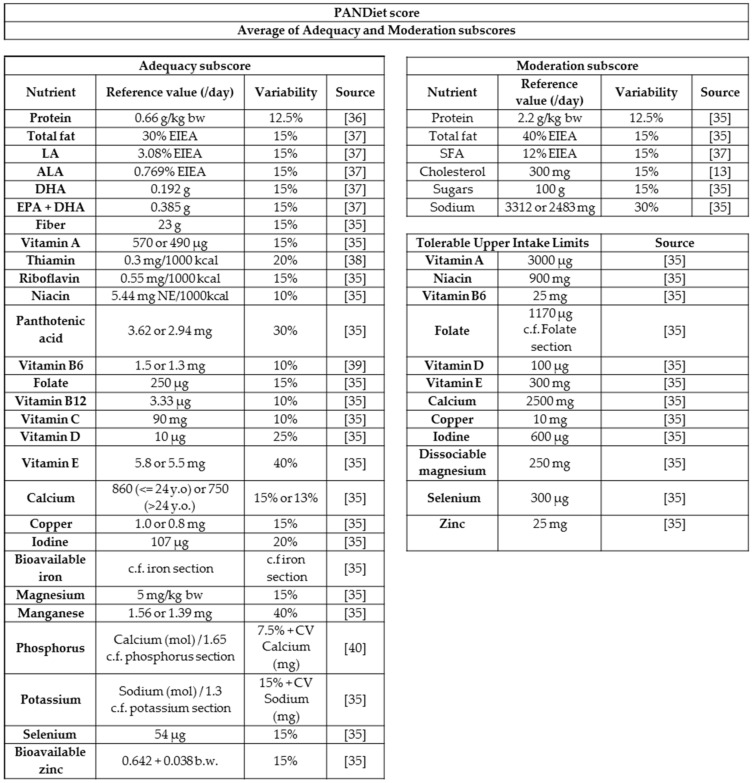
The PANDiet score, expressed as the average of an adequacy subscore (accounting for 28 nutrients), and a moderation subscore (accounting for six nutrients, plus 12 potential penalty values). DHA and EPA + DHA are weighted by ½ as DHA is counted twice. Niacin equivalents were calculated as the sum of dietary niacin and 1/60 dietary tryptophan. The upper reference value for sugars excludes lactose. The tolerable upper intake limit for vitamin A concerns retinol only. ALA, Alpha Linolenic Acid. DHA, Docosahexaenoic Acid. EIEA, Energy Intake Excluding Alcohol. EPA, Eicosapentaenoic acid. LA, Linoleic Acid. NA, Niacin Equivalent. SFA, Saturated Fatty Acid.

The nutrient adequacy of the diet was assessed using the PANDiet score [33,34], which was updated to account for the 2016 Anses guidelines [35] (Figure 1). 

As in previous versions of the PANDiet score, the Adequacy Subscore (AS) was calculated as the average probability of adequacy of nutrients for which the usual intake should be above a reference value, multiplied by 100.

For a given nutrient, the probability of adequacy was determined from the estimated average requirement (EAR) and its variability. Regarding nutrients for which no EAR was defined but an Adequate Intake (AI) had been determined, we used a pseudo-EAR calculated as follows: pseudo-EAR = AI/(1 + 2.CV), where CV is the Coefficient of Variation for the requirement, which in the absence of a specific estimated value was fixed at 15%. When the AI values were derived from the mean intake of the population, the CVs of intakes by the population were used. For iron and zinc, physiological requirements (i.e., the requirements for absorbed iron and zinc) were used because the bioavailability of these nutrients was calculated. Indeed, EARs reflect the requirements for nutrient intakes, and already account for average bioavailability. When nutrient intakes are corrected with respect to bioavailability, they must be compared with physiological requirements. For potassium and phosphorus, the requirements were dependent on their molar ratios to sodium and calcium, respectively. Thus the requirements were defined as a function of sodium and calcium molar intakes. As for iron, and the case of menstruating women, the requirements were not normally distributed so a log-normal distribution was applied. 

As in the previous version, the Moderation Subscore (MS) was calculated as the average probabilities of inadequacy of six nutrients for which an upper bound reference value exists, together with penalty values. For the six nutrients, the probability of adequacy was calculated as before, using the upper bound of the acceptable macronutrient distribution range. When no variability was specified, a value of 15% was applied. Because the reference value for sodium was the median of intake by the population, the variability of intake was used. For other vitamins and minerals with a low risk of excessive intake, a penalty value was applied: a value of 0 was added when the average intake exceeded the upper tolerable limit.

The full method is described in Methods S2. Verifications were made that the updated PANDiet score had passed the initial scheme for construct validity, as for the original version [33]. A low association with energy (r = −0.16; *p* < 0.0001), and a significant association with age, gender, energy density (*p* < 0.0001) and smoking status (*p* < 0.01) were found.

### 2.5. Diversity of the Protein Intake

A protein food diversity score (ProtDiv-S) derived from the DivS-score developed by Bianchi, Egnell et al. [41] was designed using the 43 protein groups described previously. The foods in 16 groups were composite foods, and were broken down into ingredients as described in a previous work [42]. These ingredients and the 27 other groups were allocated to the five protein groups (“Fruit and vegetables”, ”Starch”, “Legumes”, “Meat and delicatessen, fishery products and eggs” and “Milk and dairy products”) and to the 17 protein subgroups described in the PNNS nomenclature [12]. The score was then calculated as follows:

$$ProtDiv-S=\;\frac{1}{5}\times \;\overset{5}{\underset{i=1}{\sum }}\frac{Number\;of\;subgroups\;consumed\;in\;food\;group\;i}{Total\;number\;of\;subgroups\;in\;food\;group\;i}\times 100$$

### 2.6. Characterization of Dietary Patterns

The single association between possible determinants of dietary patterns and these patterns was tested under univariate analysis in order to select the relevant characteristics that should be included in logistic regression models. The variables selected were gender, age, level of education, size of town localization of the household and occupational level. After this analysis, the determinants considered were analyzed independently of the others for each dietary pattern using logistic regression models by comparison with the overall population. 

The association between probabilities of adequacy for each nutrient, AS, MS, PANDiet, ProtDiv-S and dietary patterns was assessed using ANOVA, and the difference between mean probabilities of adequacy in patterns and the overall population was assessed using ANOVA adjusted for gender. 

The Brunet algorithm was implemented using the ‘NMF’ R package [43]. All other statistical analyses were performed using SAS 9.1.3.

## 3. Results

### 3.1. Identification of Dietary Protein Patterns

A total of eight factors were identified as summarizing the protein food intake of the population using the NMF method (Table 2). Four factors concerned one major food group which contributed more than 50% to the factor (respectively poultry, pork, beef and milk), while the four others were defined by several food groups contributing less than 50%. Pearson’s correlation coefficient values were low between the different factors (≤0.34), indicating that the protein intake patterns identified were independent. 

After hierarchical cluster analysis, eight different dietary patterns were identified based on the scores of individuals for each factor. Each pattern was described by only one major factor, significantly more used in the pattern than in the overall population. The four patterns described by factors with one major contributor were identified as ‘Poultry eaters’ (9% of the population), ‘Pork eaters’ (14%), ‘Beef eaters’ (15%) and ‘Milk drinkers’ (14%). The other four were identified as ‘Processed meat eaters’, ‘Traditional eaters’, ‘Take-away eaters’ and ‘Fish eaters’ (Table 3). The “Processed meat eaters” pattern accounted for 11% of the population and concerned consumers who used the ‘Processed meat’ factor significantly more than the overall population. This factor was represented by meat dishes (e.g., paella, cassoulet or chili con carne), lamb, bread, offal, and sausages. ‘Traditional eaters’ accounted for 21% of the population and were described by the traditional factor, explained mainly by cheese, bread, fatty fish, delicatessen and eggs. The ‘Take-away eaters’ (10% of the population) used the take-away factor more than the overall population, and this was mainly characterized by take-away products such as pizza, burger, sandwiches or pancakes, and processed foods such as mixed salads and pasta dishes. Finally, ‘Fish eaters’ accounted for 6% of the population and were characterized by the ‘Fish eaters’ factor associated with fish, veal, wholemeal products, yogurts, cream cheese and soups.

**Figure 2:**
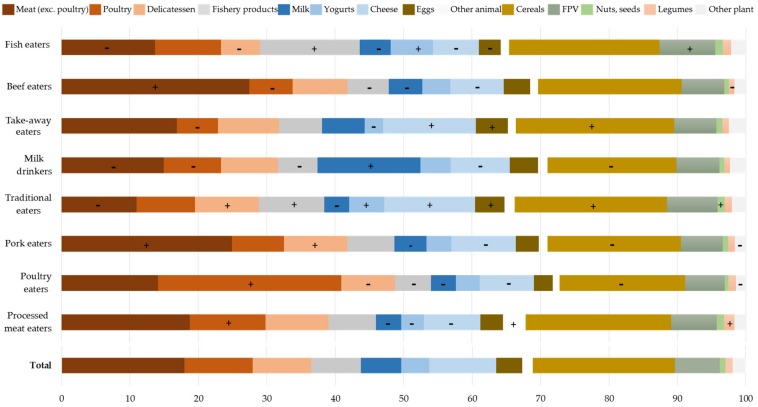
Food groups contributing to protein intake (%) in the eight clusters identified by the study. “+” means significantly superior to the mean of the overall population (*p* < 0.05) and “-“means significantly inferior to the mean of the overall population (*p* < 0.05). FPV: fruit, potatoes and vegetables.

As could be expected from the different factors, meat (without poultry) contributed more to protein intake than the overall sample in ‘Pork eaters’ and ‘Beef eaters’ (more than 25%), and less in ‘Poultry eaters’, ‘Traditional eaters’, ‘Milk drinkers’ and ‘Fish eaters’ (less than 15%; Figure 2 and Table S3). Likewise, in ‘Poultry eaters’, poultry contributed more than the overall population to protein intake (27%). Delicatessen was a high contributor to protein intake among ‘Pork eaters’ and ‘Traditional eaters’ (9%), whereas it was a low contributor for ‘Poultry eaters’ and ‘Fish eaters’ (respectively 8% and 6%). The contribution of fish to protein intake was higher among ‘Traditional eaters’ and ‘Fish eaters’ than in the overall population (respectively 10% and 15%), as was that of yogurt (6% and 5%, respectively). ‘Milk drinkers’ saw the highest contribution to protein intake for milk (15%). Cheese, eggs and cereals contributed more to protein intake among ‘Traditional eaters’ and ‘Take-away eaters’ while nuts and seeds contributed more to protein intake in ‘Traditional eaters’ (1%) and legumes in ‘Processed meat eaters’ (2%). ‘Traditional eaters’, ‘Take-away eaters’ and ‘Fish eaters’ where the patterns displayed a significantly higher percentage (~35%) of plant protein (as determined after breaking down composite dishes into ingredients), whereas poultry, pork and milk eaters had a lower percentage (~28%).

### 3.2. Characterization of Dietary Protein Patterns

The OR estimates and 95% CI values from logistic regression analyses are presented in detail in Table S1. ‘Milk drinkers’ were more likely to be women (*p* < 0.05) whereas ‘Poultry eaters’ and ‘Beef eaters’ were more likely to be men (*p* < 0.05). ‘Traditional eaters’ were more likely to be older than 50 years old than younger than 24 years old (*p* < 0.0001), which contrasted with ‘Take-away eaters’ who were more likely to be under 24 than over 50 (*p* < 0.0001). ‘Traditional eaters’ tended to be more highly educated than poorly educated (*p* < 0.05). Finally, ‘Processed meat eaters’ were more likely to live in the suburbs than in dispersed areas (*p* < 0.05) and ‘Fish eaters’ were more likely to live in villages than in dispersed areas (*p* < 0.05).

**Figure 3:**
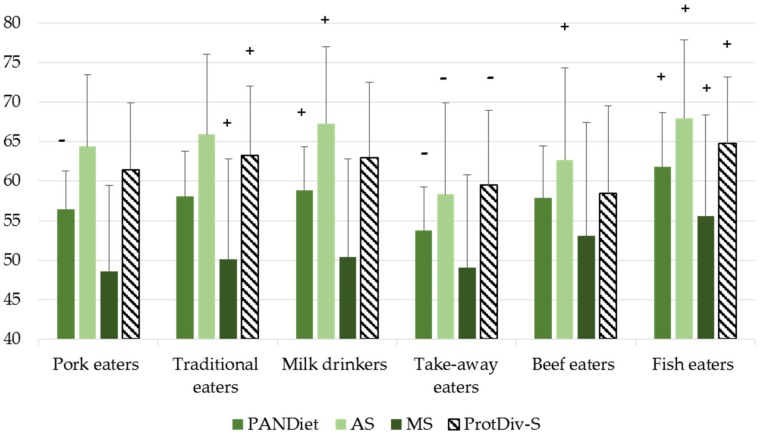
PANDiet, AS, MS and ProtDiv-S of protein dietary patterns in INCA2 study (*n* = 1678). Only those patterns which differed significantly from those of the general population for at least one parameter are shown. Significant differences are represented with a “+” if the parameter in the cluster was higher than in the overall population, and a “-“ if the parameter in the cluster was lower. AS, Adequacy Subscore. MS, Moderation Subscore. ProtDiv-S, Protein Diversity Score.

Probabilities of adequacy, PANDiet and ProtDiv-S scores for the different dietary patterns are presented in Table S2. ‘Fish eaters’ and ‘Milk eaters’ had significantly higher PANDiet scores (respectively 61.8 and 58.9) than the overall population, while those of ‘Take-away eaters’ and ‘Pork eaters’ were significantly lower (respectively 53.7 and 56.5). The AS was higher for ‘Traditional eaters’ (65.9), ‘Milk drinkers’ (67.3) and ‘Fish eaters’ (68.0), and lower for ‘Take-away eaters’ (58.3). The MS score was higher among ‘Beef eaters’ (53.1) and ‘Fish eaters’ (55.6) (Figure 3).

**Figure 4:**
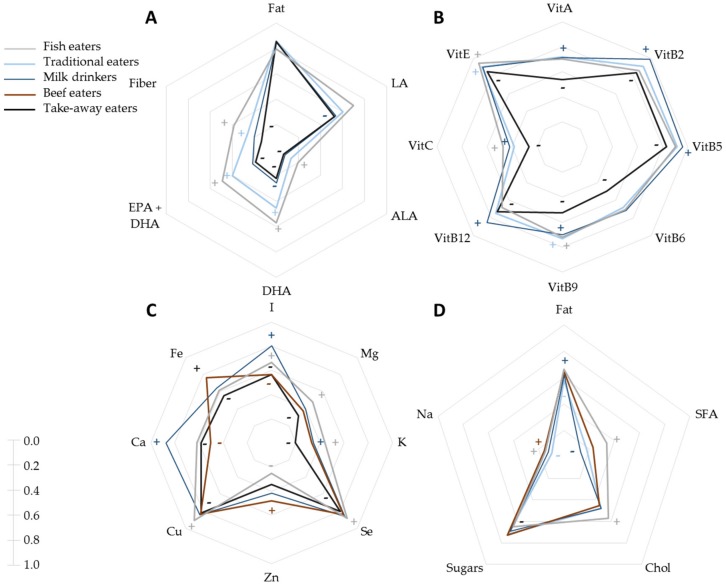
Probabilities of adequacy (PA) for nutrients in the PANDiet score. Only nutrients with a significant association between dietary patterns and PA values, and PA < 0.95 for all patterns, are shown. Significant differences between the PA of a cluster and the PA of the overall sample are represented with a “+” if the PA of the cluster is higher, and a “-“ if the PA of the cluster is lower. The four patterns with the highest number of significant differences are shown in each diagram. A: AS macronutrients. B: AS vitamins. C: AS minerals. D: MS nutrients. ALA, Alpha Linolenic Acid. Chol, Cholesterol. DHA, Docosahexaenoic Acid. EPA, Eicosapentaenoic acid. LA, Linoleic Acid. SFA, Saturated Fatty Acid. Sugars, Sugars (except lactose). Vit, Vitamin.

The PA values for fiber, EPA + DHA and DHA were higher than in the overall population among ‘Fish eaters’ and ‘Traditional eaters’, as was the PA for ALA in ‘Fish eaters’. ‘Take-away eaters’ had lower PA values for all these nutrients and LA, and ‘Milk drinkers’ had a lower probability of adequacy for DHA. (Figure 4). ‘Milk drinkers’ had higher PA values than the overall population for vitamins B1, B2, B5, B9, B12, and C, and ‘Fish eaters’ and ‘Traditional eaters’ had higher PA values for vitamins B9, E, and C (‘Fish eaters’ only). ‘Fish eaters’ had higher PA values than the overall population for iodine, magnesium, potassium, selenium, manganese and copper, but a lower PA for zinc. ‘Beef eaters’ had higher PA values for zinc and iron, but lower values for iodine and calcium. ‘Traditional eaters’ had higher PA values for copper, manganese and calcium, but lower values for iron, zinc and potassium. ‘Milk drinkers’ had higher PA values for iodine, potassium and calcium, but lower values for manganese. ‘Take-away eaters’ had lower PA values for all vitamins and minerals except from zinc, phosphorus and vitamin B3. ‘Fish eaters’ had higher PA values for SFA, cholesterol and sodium. ‘Milk drinkers’ had a lower PA value for SFA, ‘Poultry eaters’ for cholesterol and ‘Traditional eaters’ for sodium. 

Finally, dietary protein patterns displayed varied degrees of diversity of protein foods: healthy and ‘Traditional eaters’ had a higher ProtDiv-S score than the overall population (respectively 70.1 and 71.4) whereas ‘Take-away eaters’ and ‘Beef eaters’ had lower scores (respectively 63.7 and 63.9). 

## 4. Discussion

During this study we showed that the general adult population can be clearly discriminated in terms of its protein intake profile. The eight dietary protein patterns are very easy to interpret and have different nutritional characteristics, as shown by the probabilities of adequacy of each nutrient intake (PA), overall nutrient adequacy (PANDiet score) and protein source diversity. The most important contrasts regarding the PANDiet score and protein diversity were found between the ‘Fish eaters’ pattern and the ‘Take-away eaters’ pattern. Whereas the overall nutrient adequacy of the diets of other protein patterns were similar, there were differences in the adequacy of intake of many individual nutrients, showing that these protein patterns, which are significant in the underlying structure of the diet, are associated with specific clusters of nutrient intake.

### 4.1. Identification of Dietary Protein Patterns

Dietary protein patterns were identified using the NMF method and not standard methods such as Principal Component Analysis, Factor Analysis or clustering (Hierarchical Cluster Analysis, k-means) [15,44]. We made this choice because these latter methods have limitations when applied to food intake data because of to the significant number of zeros and non-negative data, as acknowledged in recent publications on identifying food patterns [16,29,30]. As no consensus has been reached regarding the choice of algorithm to use when identifying dietary patterns NMF, we chose the Brunet algorithm, although we ran the NMF method with other algorithms and found similar results. The choice of protein food groups to be included in order to identify dietary patterns was important as these results are known to be strongly affected by the groups employed [16,45]. The 43 groups used were consistent with those applied in a previous study of dietary protein clusters [18].

Because the eight dietary patterns were identified using protein food groups only, they were not comparable to other dietary pattern studies based on overall food groups. The only other studies to have identified dietary patterns from protein food groups were performed in the US by Mangano et al. [17,18]. Of the five and six of the patterns that were identified during these studies, respectively, five were quite similar to those we found: (‘Chicken’, ‘Fish’, ‘Processed foods’, ‘Red meat’ and ‘Low-fat milk’) and one was different (‘Legumes’ pattern). Their ‘Chicken’, ‘Red meat’ and ‘Low-fat milk’ patterns were consistent with the ‘Poultry’, ‘Beef’ and ‘Milk’ eaters determined in the present study, with the protein intake from chicken, beef and milk, respectively, being significantly higher than in the general population. Our ‘Milk drinkers’ pattern included all types of milk (not just low-fat milk) because a large majority (85%) of the milk consumed in this French population is semi-skimmed (data not shown). The Fish pattern described by Mangano et al. [17,18] corresponded to the ‘Fish eaters’ pattern in our study, as fish, fruits and vegetables also contributed significantly more to protein intake in this pattern than in the overall population. The Fast Food/Processed Food pattern corresponded to our ‘Take-away eaters’, characterized by a major contribution to protein intake of take-away and processed foods. Their Legumes pattern had no equivalent in our study. Finally, the ‘Traditional eaters’ in our study were not identified in the US study, and may be more specific to the French diet. Indeed, they were characterized by an important contribution of cheese, delicatessen, bread and eggs to protein intake, as has been described in other French studies [16,46]. 

### 4.2. Characterization of Dietary Patterns

Dietary patterns were characterized by determining their association with nutrient adequacy. The PANDiet score was updated to account for the most recent nutrient references published by the French authority in 2016 [35]. The probability approach applied to the PANDiet score enabled an accurate assessment of individual PAs, particularly for nutrients with specific distributions such as iron in menstruating women. Moreover, because the bioavailability of protein, iron and zinc was taken into account, this approach could generate the most precise PA estimates. It should be noted that the data for phytate, the percentage of heme iron and amino acids were adapted from different sources, but the mean bioavailability and prevalence of adequacy for zinc and iron were consistent with other studies, which had used different data [47]. Furthermore, as no biological data about individual serum ferritin levels were available, we fixed a cut-off value of 15 mg/L corresponding to a lack of stored iron. Although this hypothesis would overestimate fractional absorption, it still accounted for differences in iron PA between the dietary patterns. 

The patterns had clearly different PANDiet scores and thus differed in terms of the overall nutrient adequacy of the diets. However, the PANDiet scores of these patterns were not explained by the same nutrients. Indeed, ‘Fish eaters’ and ‘Milk drinkers’ had higher PANDiet scores than the overall population, but this could be explained by both a higher AS and MS in ‘Fish eaters’, but only a higher AS among ‘Milk drinkers’. ‘Milk drinkers’ had high PA values for vitamins, iodine and calcium but low PAs for SFA and DHA, whereas ‘Fish eaters’ had high PAs for most nutrients except zinc. Conversely, ‘Pork eaters’ and ‘Take-away eaters’ had the lowest PANDiet scores, which could be explained by a lower AS in ‘Take-away eaters’ while the AS and MS among ‘Pork eaters’ were not significantly lower than in the overall population. ‘Take-away eaters’ had lower PAs for most nutrients, while ‘Pork eaters’ had significantly lower PAs for just a few nutrients (Vitamin B12, Calcium, SFA). Some patterns displayed higher PAs for certain nutrients that are closely associated with the major food contributing to protein intake: thus ‘Traditional eaters’ and ‘Milk drinkers’ had high PA values for calcium that could be attributed to their cheese and milk intakes, ‘Traditional eaters’ and ‘Fish eaters’ had high PAs for EPA and EPA + DHA which falls in line with their fish intake, and ‘Beef eaters’ had high iron and zinc intakes from red meat. However, PA values were not always associated with the main food contributing to protein intake: contrary to what might have been expected given the SFA composition of cheese and delicatessen, ‘Traditional eaters’ did not have lower PA values for SFA than the overall population. Consequently, it appeared that the nutrient profiles of these dietary patterns were also the result of complex food associations that could be complementary.

The diversity of protein sources varied between dietary patterns, but patterns with higher PANDiet scores than the overall population did not all have higher ProtDiv-S scores than the overall population. Indeed, ‘Milk drinkers’ had a higher PANDiet score but not a higher ProtDiv-S score, whereas the opposite was true for ‘Traditional eaters’. This could be explained by the significant (*p* < 0.001) but small associations between the ProtDiv-S and PANDiet scores (β = 0.15), AS (β = 0.3) and MS (β = −0.15).

## 5. Conclusions

Protein patterns could clearly be identified in the general population and they were associated with socio-demographic characteristics, nutrient adequacy and dietary diversity of protein sources. The ‘Fish eaters’ pattern was found to be associated with high overall nutrient adequacy and protein diversity, whereas the ‘Take-away eaters’ pattern was associated with lower nutrient adequacy and protein diversity. The other patterns were associated with nutrient adequacy that was often linked to the nutrient profile of the food groups contributing most to the interpretation of a protein pattern. A major conclusion of this study is that protein patterns constitute strong elements in the background structure of the dietary intake of a general population and that these patterns are associated with the nutrient profile that they convey. We conclude that protein patterns are important to public nutrition, because protein choices can markedly shape nutrient intake and resulting dietary quality. The manipulation of protein intake could have significant and complex effects on nutrient quality in the general population.

## Figures and Tables

**Table 1:** Protein food sub-groups selected to identify dietary protein patterns and number of protein foods in each sub-group.

Plant Protein Sub-Groups		Animal Protein Sub-Groups		Composite Protein Subgroups	
Protein Sub-Groups	No. of Protein Food	Protein Sub-Groups	No. of Protein Food	Protein Sub-Groups	No. of Protein Food
Bread	13	Beef	16	Pizza	8
Crispbread	2	Pork	5	Quiche	5
Wholemeal bread or crispbread	2	Veal	6	Pastry rolls	4
Pastas	3	Lamb	4	Burgers	4
Cooked wheat	2	Poultry	18	Sandwiches	17
Beans and peas	2	Offal	15	Other sandwiches	8
Legumes	8	Ham	16	Soups	6
Seeds and nuts	6	Sausage	24	Meat dishes	18
		Pâté	11	Pasta or potato dishes	11
		Lean fish	31	Pancakes	13
		Fatty fish	30	Dishes without filling	14
		Fish derivatives	9	Vegetables dishes	5
		Shellfish	16	Mixed salads	5
		Milk	8	Custards	5
		Cocoa beverages	3	Desserts	8
		Sweetened yogurts	14		
		Natural yogurts	7		
		Cream cheese	15		
		Cheese	90		
		Eggs	11		

**Table 2:** Factors identified applying the NMF method to the INCA2 study data (*n* = 1678) and loadings of protein food subgroups (%) on the factors.

Processed Meat		Poultry		Pork		Traditional	
Protein Food Subgroup	Loading (%)	Protein Food Subgroup	Loading (%)	Protein Food Subgroup	Loading (%)	Protein Food Subgroup	Loading (%)
Meat dishes ^1^	38.2	Poultry	83.8	Pork	62.1	Cheese	26.1
Lamb	13.2	Bread	4.6	Bread	11.0	Bread	25.1
Bread	12.8	Yogurts	3.0	Sausage	7.3	Fatty fish	9.3
Offal	10.3			Ham	3.1	Ham	7.3
Sausages	6.9			Pâté	2.9	Eggs	4.9
Cheese	6.9			Pasta	2.7	Sausage	4.7
						Natural yogurts	4.0
						Cream cheese	2.6
**Milk**		**Take-Away**		**Beef**		**Fish**	
**Protein Food Subgroup**	**Loading (%)**	**Protein Food Subgroup**	**Loading (%)**	**Protein Food Subgroup**	**Loading (%)**	**Protein Food Subgroup**	**Loading (%)**
Milk	64.7	Pizza	23.2	Beef	69.1	Lean fish	38.6
Cocoa beverages	8.5	Other sandwiches	10.9	Bread	9.6	Veal	17.6
Yogurts	2.9	Burgers	10.7	Pasta	4.5	Wholemeal bread	8.6
Pasta dishes	2.9	Mixed salads	9.4	Ham	4.3	Natural yogurts	5.3
Cream cheese	2.7	Sandwiches	8.7	Sausages	3.3	Yogurts	4.0
		Pasta dishes	7.1	Yogurts	3.2	Cream cheese	3.3
		Cheese	5.2			Soups	2.7
		Pancakes	4.2				
		Pasta	3.1				
		Sausages	2.9				

^1^ Loading of protein food subgroup on the factor ≥2.5%.

**Table 3:** Dietary protein patterns identified from the scores of individuals from the INCA2 study (*n* = 1678) for the different factors.

Dietary Protein Pattern ^1^	No. of Individuals in the Pattern	Factors Contributing to the Pattern ^2^	% Contribution of the Factor
Processed meat eaters	192	Processed meat	36
Poultry eaters	144	Poultry	44
Pork eaters	239	Pork	36
Traditional eaters	347	Traditional	38
Milk drinkers	241	Milk	28
Take-away eaters	172	Take-away	37
Beef eaters	244	Beef	37
Fish eaters	99	Fish	36

^1^ Patterns identified from hierarchical cluster analysis on the scores of each individual for the eight factors. Each pattern was described by only one major factor, used significantly more in the pattern than in the overall population. ^2^ These factors contributed significantly more to the pattern than to the overall population.

## Supplementary Materials

The following are available online at http://www.mdpi.com/2072-6643/10/2/226/s1, Methods S1: Bioavailability of some nutrients, Methods S2: Reference values used to estimate the probabilities of adequacy of the components of the PANDiet score, Table S1: Odd-ratios (OR) [95% CI] and p-values of socio-demographic determinants of dietary patterns, Table S2: Mean (±SD) probabilities of adequacy for components of the PANDiet score and ProtDiv-S, Table S3. Mean (±SD) contribution of food groups to protein intake.

## References

[1] Nijdam D., Rood T., Westhoek H. (2012). The price of protein: Review of land use and carbon footprints from life cycle assessments of animal food products and their substitutes. Food Policy.

[2] Clarys P., Deliens T., Huybrechts I., Deriemaeker P., Vanaelst B., De Keyzer W., Hebbelinck M., Mullie P. (2014). Comparison of nutritional quality of the vegan, vegetarian, semi-vegetarian, pesco-vegetarian and omnivorous diet. Nutrients.

[3] Mullee A., Vermeire L., Vanaelst B., Mullie P., Deriemaeker P., Leenaert T., De Henauw S., Dunne A., Gunter M.J., Clarys P. (2017). Vegetarianism and meat consumption: A comparison of attitudes and beliefs between vegetarian, semi-vegetarian, and omnivorous subjects in belgium. Appetite.

[4] Derbyshire E.J. (2016). Flexitarian diets and health: A review of the evidence-based literature. Front. Nutr..

[5] Conrad Z., Karlsen M., Chui K., Jahns L. (2017). Diet quality on meatless days: National health and nutrition examination survey (NHANES), 2007–2012. Public Health Nutr..

[6] Tonstad S., Clifton P., Mariotti F. (2017). 20 - vegetarian diets and the risk of type 2 diabetes. Vegetarian and Plant-Based Diets in Health and Disease Prevention.

[7] Mann J., Mariotti F. (2017). 23-ischemic heart disease in vegetarians and those consuming a predominantly plant-based diet. Vegetarian and Plant-Based Diets in Health and Disease Prevention.

[8] Dinu M., Abbate R., Gensini G.F., Casini A., Sofi F. (2017). Vegetarian, vegan diets and multiple health outcomes: A systematic review with meta-analysis of observational studies. Crit. Rev. Food Sci. Nutr..

[9] Millward D.J. (1999). The nutritional value of plant-based diets in relation to human amino acid and protein requirements. Proc. Nutr. Soc..

[10] Tome D. (2013). Digestibility issues of vegetable versus animal proteins: Protein and amino acid requirements–functional aspects. Food Nutr. Bull..

[11] Phillips S.M., Fulgoni V.L., Heaney R.P., Nicklas T.A., Slavin J.L., Weaver C.M. (2015). Commonly consumed protein foods contribute to nutrient intake, diet quality, and nutrient adequacy. Am. J. Clin. Nutr..

[12] Anses (2016). Updating of the PNNS guidelines: Revision of the food-based dietary guidelines. Anses Opinion—Collective Expert Report.

[13] Dietary Guidelines Advisory Committee (2010). Report of the Dietary Guidelines Advisory Committee on the Dietary Guidelines for Americans, 2010.

[14] Wirfält E., Drake I., Wallström P. (2013). What do review papers conclude about food and dietary patterns?. Food Nutr. Res..

[15] Newby P., Tucker K.L. (2004). Empirically derived eating patterns using factor or cluster analysis: A review. Nutr. Rev..

[16] Gazan R., Béchaux C., Crépet A., Sirot V., Drouillet-Pinard P., Dubuisson C., Havard S. (2016). Dietary patterns in the French adult population: A study from the second French national cross-sectional dietary survey (inca2) (2006–2007). Br. J. Nutr..

[17] Mangano K.M., Sahni S., Kiel D.P., Tucker K.L., Dufour A.B., Hannan M.T. (2017). Dietary protein is associated with musculoskeletal health independently of dietary pattern: The Framingham third generation study. Am. J. Clin. Nutr..

[18] Mangano K.M., Sahni S., Kiel D.P., Tucker K.L., Dufour A.B., Hannan M.T. (2015). Bone mineral density and protein-derived food clusters from the Framingham offspring study. J. Acad. Nutr. Diet..

[19] de Gavelle E., Huneau J.-F., Bianchi M.C., Verger O.E., Mariotti F. (2017). Protein adequacy is primarily a matter of protein quantity, not quality: Modeling an increase in plant: Animal protein ratio in French adults. Nutrients.

[20] Dubuisson C., Lioret S., Touvier M., Dufour A., Calamassi-Tran G., Volatier J.-L., Lafay L. (2010). Trends in food and nutritional intakes of French adults from 1999 to 2007: Results from the inca surveys. Br. J. Nutr..

[21] Amirabdollahian F., Ash R. (2010). An estimate of phytate intake and molar ratio of phytate to zinc in the diet of the people in the United Kingdom. Public Health Nutr..

[22] Centre d’Information des Viandes & INRA Valeurs Nutritionnelles des Viandes. http://www.lessentieldesviandes-pro.org/pdf/PDF-tous%20morceaux.pdf.

[23] Centre d’Information des Viandes INAPORC: Etude Nutritionnelle de la Viande de Porc Fraiche. http://www.lessentieldesviandes-pro.org.

[24] Kongkachuichai R., Napatthalung P., Charoensiri R. (2002). Heme and nonheme iron content of animal products commonly consumed in Thailand. J. Food Compost. Anal..

[25] Armah S.M., Carriquiry A., Sullivan D., Cook J.D., Reddy M.B. (2013). A complete diet-based algorithm for predicting nonheme iron absorption in adults. J. Nutr..

[26] Hallberg L., Hulthen L. (2000). Prediction of dietary iron absorption: An algorithm for calculating absorption and bioavailability of dietary iron. Am. J. Clin. Nutr..

[27] Miller L.V., Krebs N.F., Hambidge K.M. (2007). A mathematical model of zinc absorption in humans as a function of dietary zinc and phytate. J. Nutr..

[28] Grieger J.A., Scott J., Cobiac L. (2011). Dietary patterns and breast-feeding in Australian children. Public Health Nutr..

[29] Sy M.M., Feinberg M., Verger P., Barré T., Clémençon S., Crépet A. (2013). New approach for the assessment of cluster diets. Food Chem. Toxicol..

[30] Zetlaoui M., Feinberg M., Verger P., Clémençon S. (2011). Extraction of food consumption systems by nonnegative matrix factorization (nmf) for the assessment of food choices. Biometrics.

[31] Brunet J.-P., Tamayo P., Golub T.R., Mesirov J.P. (2004). Metagenes and molecular pattern discovery using matrix factorization. Proc. Natl. Acad. Sci. USA.

[32] Kaufman L., Rousseeuw P.J. (2009). Finding Groups in Data: An Introduction to Cluster Analysis.

[33] Verger E.O., Mariotti F., Holmes B.A., Paineau D., Huneau J.-F. (2012). Evaluation of a diet quality index based on the probability of adequate nutrient intake (pandiet) using national French and us dietary surveys. PLoS ONE.

[34] Bianchi C.M., Mariotti F., Verger E.O., Huneau J.-F. (2016). Pregnancy requires major changes in the quality of the diet for nutritional adequacy: Simulations in the French and the United States populations. PLoS ONE.

[35] Anses (2016). Actualisation des repères du PNNS: Élaboration des références nutritionnelles. Rapport d'expertise collective.

[36] FAO Expert Consultation (2011). Dietary protein quality evaluation in human nutrition. FAO Food Nutr. Pap..

[37] Anses (2011). Actualisation des apports nutritionnels conseillés pour les acides gras. Rapport d’expertise collective.

[38] EFSA Panel on Dietetic Products, Nutrition and Allergies (NDA) (2016). Scientific opinion on dietary reference values for thiamin. EFSA J..

[39] EFSA Panel on Dietetic Products, Nutrition and Allergies (NDA) (2016). Scientific opinion on dietary reference values for vitamin b6. EFSA J..

[40] EFSA Panel on Dietetic Products, Nutrition and Allergies (NDA) (2015). Scientific opinion on dietary references values for phosphorus. EFSA J..

[41] Bianchi C.M., Egnell M., Huneau J.-F., Mariotti F. (2016). Plant protein intake and dietary diversity are independently associated with nutrient adequacy in French adults. J. Nutr..

[42] Camilleri G.M., Verger E.O., Huneau J.-F., Carpentier F., Dubuisson C., Mariotti F. (2013). Plant and animal protein intakes are differently associated with nutrient adequacy of the diet of French adults. J. Nutr..

[43] Gaujoux R., Seoighe C. (2010). A flexible r package for nonnegative matrix factorization. BMC Bioinform..

[44] Quatromoni P., Copenhafer D., Demissie S., D'Agostino R., O'Horo C., Nam B., Millen B. (2002). The internal validity of a dietary pattern analysis. The Framingham nutrition studies. J. Epidemiol. Commun. Health.

[45] McCann S.E., Marshall J.R., Brasure J.R., Graham S., Freudenheim J.L. (2001). Analysis of patterns of food intake in nutritional epidemiology: Food classification in principal components analysis and the subsequent impact on estimates for endometrial cancer. Public Health Nutr..

[46] Mathe T., Francou A., Colin J., Hebel P. (2011). Comparaison des modèles alimentaires français et états-uniens. Cahier de recherche du CREDOC.

[47] Perignon M., Barré T., Gazan R., Amiot M.-J., Darmon N. (2018). The bioavailability of iron, zinc, protein and vitamin a is highly variable in French individual diets: Impact on nutrient inadequacy assessment and relation with the animal-to-plant ratio of diets. Food Chem..

