# Supplementary material for: Patterns of Protein Food Intake Are Associated with Nutrient Adequacy in the General French Adult Population
Source: Nutrients. 2018 Feb 17;10(2):226. doi: 10.3390/nu10020226 (PMC5852802; doi:10.3390/nu10020226)
Supplement: Supplementary file 1 [file nutrients-10-00226-s001.zip › OSM.docx]

**Methods S1: Bioavailability of some nutrients**

As underlined by some authors [[1-3](#_ENREF_1)], bioavailability of many nutrients varies between food sources, and particularly between animal and plant sources. Recent literature has reported factors influencing bioavailability for nutrients like protein, zinc, iron, calcium of vitamin A.

**Vitamin A**

Vitamin A intake comes from two majors sources: preformed vitamin A (retinol) from animal sources and provitamin A carotenoids, such as β-carotene, from plant sources. As reported by the IOM and EFSA, many conversion factors have been determined to express β-carotene as a retinol equivalent. These factors are highly dependent on individual related factors such as genetics, age, sex, nutritional status, or digestive dysfunctions, as well as food processing or food matrix [[4](#_ENREF_4),[5](#_ENREF_5)].

Against this background, it was considered that data on conversion factors for some specific foods could not be used as it was dependent on individual related factors and food processing and matrix. We chose to use the IOM’s 12:1 conversion factors for β-carotene to retinol equivalent for all food items [[5](#_ENREF_5)].

**Calcium**

Studies showed that calcium absorption was similar for the major food contributing to its intake. However, foods rich in oxalic and phytic acid lower calcium absorption, which varies also depending the time of consumption and the amount consumed. Furthermore, sodium and protein intake are consider to influence calcium requirements [[6](#_ENREF_6)].

As available data did not provide any predictive equation to estimate the link between oxalic and phytic acid, protein or sodium intake and calcium bioavailability, we considered bioavailability to be similar in all food items.

**Zinc**

Phytate intake was found to be the major limitation of zinc absorption. A mathematical model was used to predict zinc absorption as a function of dietary zinc and phytate intakes [[7](#_ENREF_7)]:

$$TAZ=0.5 \times\left\{ 0.13+TDZ+ 0.10\left( 1+\frac{TDP}{1.2} \right)- \sqrt{\left( 0.13+TDZ+0.10(1+\frac{TDP}{1.2}) \right)^{2}-4\times0.13\times TDZ} \right\}$$

TAZ: total absorbed zinc (mmol), TDZ: total dietary zinc (mmol) and TDP: total dietary phytate (mmol).

**Iron**

Iron intakes comes from two majors forms: heme and nonheme iron. Heme iron is only found in animal sources. Nonheme iron absorption mainly depends on the nutritional status of individuals, but also on dietary factors such as vitamin C, meat, fish and poultry, tea, phytate and calcium intakes. We used the following equation to predict nonheme iron absorption [[8](#_ENREF_8)]:

$$Ln Absorption \left( \% \right)= 6.294 - 0.709 ln \left( \mathrm{SF} \right)+ 0.119 ln \left( C \right)+ 0.006 ln \left( MFP + 0.1 \right)-0.055\ln\left( T+0.1 \right)-0.247\ln\left( P \right)-0.137\ln\left( Ca \right)-0.083 ln(NH)$$

Heme iron Absorption was calculated from the following equation [[9](#_ENREF_9)]:

$$Log Absorption \left( \% \right)= 1.9897 - 0.3092 \times log (SF)$$

where SF is serum ferritin (mg/L), C is vitamin C (mg), MFP is meat, fish, and poultry (g), T is tea (number of cups), P is phytate (mg), Ca is calcium (mg), and NH is nonheme iron (mg).

As no data was available on serum ferritin of individuals, it was set to the cut off value for low stores of 15 mg/L. Although this hypothesis overestimates the absorption, it does not impact the relative PANDiet score between dietary patterns.

**Protein**

Protein quality depends on two major factors: the digestibility and the amino acid profile. Both factors are taken into account be the Digestible Indispensable Amino Acid Score (DIAAS) developed by the FAO [[10](#_ENREF_10)]. As there is no inadequate intake of amino acids for any of the individuals in the initial situation (de Gavelle et al, 2017), there is no need to use an amino acid score.

To take into account the lower digestibility of plant proteins, a penalty of 5% was applied to protein intake from plant protein food items. This coefficient was calculated from the true ileal protein digestibility studies in humans by calculating the difference between the mean digestibility of studies with plant protein food diets and the mean digestibility of studies with animal protein food diets, weighted by the sample size in studies [[11](#_ENREF_11),[12](#_ENREF_12)].

**Methods S2.** Reference values used to estimate the probabilities of adequacy of the components of the PANDiet score

Like the method set in the 2016 Anses opinion, if an EAR from an EFSA opinion was more recent than the Anses opinion, we used this value instead of the 2016 Anses value.

**Protein**

We used the value of 0.66g/kg bw endorsed by Anses, which is adapted to each individual, rather than the lower bound of the acceptable distribution range expressed as percent of energy intake.

Likewise, we used the value of the 2,2g/kg bw as an upper bound value used to set the upper bound of the acceptable distribution range.

**Total fat and fatty acids**

In the Anses opinion, the lower bound reference value for total was set to 35% of the EIEA to ensure an adequate intake of DHA. However, the opinion also stated that the minimal requirement is 30% of the EIEA. As EPA and DHA intakes are already taken into account in the PANDiet score, it was decided to use the 30% value to set the reference intake for the PANDiet calculation.

EAR for LA, ALA, DHA, DHA + EPA were calculated from the 2011 Anses opinion by dividing the AI by 1.3 (considering a variability of 15%).

The upper bound value of 40% of the EIEA of the Anses opinion was used in this study, and the value of 12% of EIEA for saturated fatty acids of the 2011 opinion was used.

Anses did not set any upper bound value for cholesterol, but it was decided to set the value of 300mg/d previously used by the IOM [[13](#_ENREF_13)].

**Carbohydrates**

It was decided that the values for the acceptable distribution range for carbohydrate intake would not be included in the PANDiet score because this estimation was based of the reference values for protein, fibres and total fat intakes, which are already taken into account in the PANDiet.

The upper bound value of 100g/d for sugars (except from lactose) was used in this study. The lactose intake was estimated as described in the Anses opinion.

**Fibers**

A pseudo-EAR was calculated from the Adequate Intake of 30g/d published by Anses and a standard variability of 15% and set to 23g/d.

**Vitamin A**

The EAR of 570 or 490 µg/d and the variability of 15% set by Anses was used in this study, as well as the upper bound value of 3000 µg for retinol only.

**Thiamin**

EFSA published an opinion on the EAR for thamin after the Anses opinion [[14](#_ENREF_14)]. It was decided to use the value of 0.3 mg/1000 kcal (20%) set by EFSA.

**Riboflavin**

A pseudo EAR was calculated from the Adequate Intake of 0.71MJ/1000kcal (15%) published by Anses and set to 0.55mg/1000kcal.

**Niacin**

The EAR of 5.44mg of niacin equivalent /1000kcal/d (10%) set by Anses was used in this study. To calculate the niacin equivalent intake we added the niacin intake with 1/60e of the tryptophan intake as described in the opinion. The upper bound value was set to 900 mg of NE, considering that the whole niacin intake was nicotinamide.

**Pantothenic acid**

The Anses opinion set an AI of 5.8 (men) or 4.7 mg/d (women) of pantothenic acid based on the mean intake in the INCA2 population. It was decided to calculate a pseudo-EAR based on a variability of 30% corresponding to the variability of intake in INCA2. Thus, for an intake of 5.8 or 4.7 mg/d, individuals have a 2.5% probability of inadequate intake.

The pseudo-EAR calculated was 3.62 or 2.94 mg/d (30%).

**Vitamin B6**

EFSA published an opinion on the EAR for vitamin B6 after the Anses opinion [[15](#_ENREF_15)]. It was decided to use the value of 1.5 or 1.3 mg/d (10%) set by EFSA. The upper bound limit of 25mg/d set in the Anses opinion was used in this study.

**Folate**

The EAR of 250 µg/d and the variability of 15% set by Anses was used in this study. The upper bound value was set to 1170 by adding the 5e percentile of intake to the value set by Anses, considering that the lower folate consumers did not consume any folic acid.

**Vitamin B12**

A pseudo EAR was calculated from the AI of 4 µg/d considering a variability of 10% as in the previous Anses opinion. Thus the pseudo-EAR was set to 3.33 µg/d (10%).

**Vitamin C**

The EAR of 90 mg/d and the variability of 10% set by Anses was used in this study.

**Vitamin D**

The EAR of 10 µg/d and the variability of 25% set by Anses was used in this study, as well as the upper bound value of 100 µg/d.

**Vitamin E**

The Anses opinion set an AI of 10.5 (men) or 9.9 (women) mg/d of vitamin E based on the mean intake in the INCA2 population. It was decided to calculate a pseudo-EAR based on a variability of 40% corresponding to the variability of intake in INCA2. Thus, for an intake of 10.5 or 9.9 mg/d, individuals have a 2.5% probability of inadequate intake.

The pseudo-EAR calculated was 5.8 or 5.5 mg/d (40%).

**Calcium**

The EAR of 860 mg/d for individuals younger than 24 y.o. or 750 g/d set by Anses was used in this study. The variability of 13% was used for individuals > 24 y.o. as it was calculated from balance studies. As no variability was set for young adults, we considered the standard variability of 15%. The upper bound value of 2500 mg/d set by Anses was used.

**Copper**

The EAR of 1 or 0.8 mg/d and the variability of 15% set by Anses was used in this study, as well as the upper bound limit of 10 mg/d.

**Iodine**

No EAR was set by Anses, thus a pseudo-EAR was calculated from the AI of 150 µg/d considering a variability of 20%, as set by the IOM from balance studies. The upper bound value was set to 600µg/d like in the Anses opinion.

**Iron**

We compared bioavailable iron intake to physiological requirements, as the bioavailability of iron was taken into account in this study.

For men and non-menstruating women, we used the raw EAR values used by Anses to calculate the 6 mg/d EAR. Thus we considered an EAR of 0.95 mg/d and a variability of 40%.

For menstruating women, as the distribution of their requirements is not normal, we modelled the distribution using a lognormal function, by adding the basal iron losses (normally distributed) to the menstrual losses (exponentially distributed, λ=ln(2)/0.28), as detailed in the Anses opinion, with a Monte-Carlo simulation on 1000 individuals. The combination of basal and menstrual losses followed a lognormal distribution (p = 0.15).


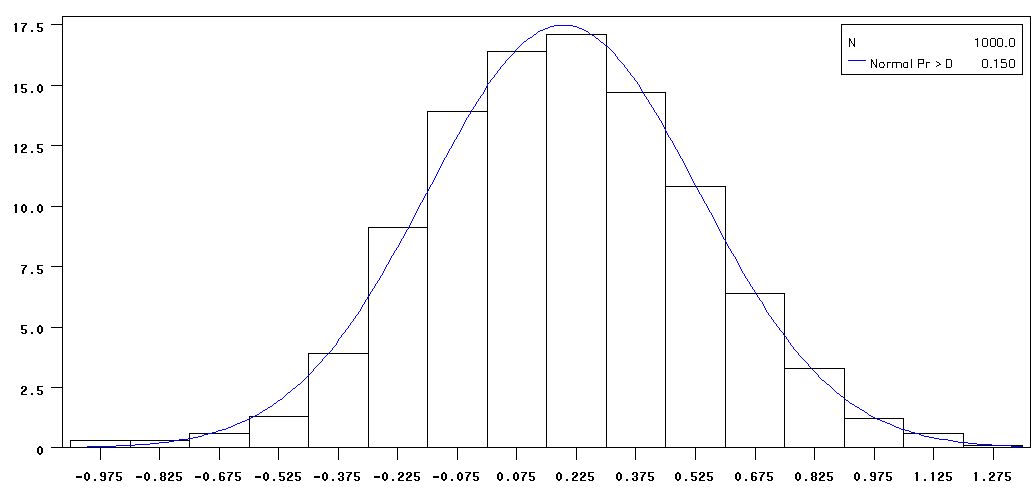


Ln (total iron losses)

Thus it was considered that the logarithm of the physiological requirements followed a normal distribution (µ=0.18, σ=0.34).

**Magnesium**

The AI set in the Anses opinion resulted from the 2001 Anses opinion where an EAR of 5 mg/kg b.w/d was defined. It was decided to use this value as it was more precise for an individual approach. However it was decided to set the variability to the standard variability of 15% as the 10% used previously corresponded the standard variability of the IOM.

It was considered that only Mg from mineral water was dissociable Mg, thus the upper bound value of 250mg/d was set only from Mg from mineral water.

**Manganese**

The Anses opinion set an AI of 2.8 or 2.5 mg/d of manganese based on the mean intake in the INCA2 population. It was decided to calculate a pseudo-EAR based on a variability of 40% corresponding to the variability of intake in INCA2. Thus, for an intake of 1.5 or 1.4 mg/d, individuals have a 2.5% probability of inadequate intake.

The pseudo-EAR calculated was 1.5 or 1.4 mg/d (40%).

**Phosphorus**

Anses decided to follow the EFSA opinion on phosphorus dietary values [[16](#_ENREF_16)]. In this opinion, EFSA considered a calcium to phosphorus molar ratio of 1.4:1 and the AI of calcium to set the reference value of 550 mg/d.

It is also stated that the calcium to phosphorus molar ratio in the body ranges from 1.9:1 to 1.4:1.

In order to have a 2.5% of inadequate intake in the population for a 1.4:1 molar ratio (which is the method used by EFSA), and a 97.5% inadequate intake for a 1.9:1 molar ratio, it was decided to set the pseudo molar EAR to the molar calcium intake divided by 1.65, with a variability of 7.5%.

The formula used to assess the probability of inadequacy was:

$F(\frac{P\left( mol/d \right) - \frac{Ca\left( mol/d \right)}{1.65}}{\sqrt{\left( 7.5\%\times\frac{Ca\left( mol/d \right)}{1.65} \right)^{2}+{{SD}_{Ca}}^{2}/n+{SD}_{P}^{2}/n}}$)

F was “probnorm” function in SAS

P(mol/d) was the mean phosphorus molar intake

Ca(mol/d) was the mean calcium molar intake

SD_P_² was the interindividual variability of P intake

SD_Ca_² was the interindividual variability of Ca intake

**Potassium**

Anses considered that a sodium to potassium molar ratio of 1:1 was adequate for the population. Thus we calculated a pseudo molar EAR by dividing the sodium intake by 1.3, considering a standard 15% variability of requirement. Thus, an individual with a molar ratio of 1:1 would have a 2.5% probability of inadequate intake.

The formula used to assess the probability of inadequacy was:

$F(\frac{K\left( mol/d \right) - \frac{Na\left( mol/d \right)}{1.3}}{\sqrt{\left( 15\%\times\frac{Na\left( mol/d \right)}{1.3} \right)^{2}+{{SD}_{Na}}^{2}/n+{SD}_{K}^{2}/n}}$)

F was “probnorm” function in SAS

K(mol/d) was the mean potassium molar intake

Na(mol/d) was the mean sodium molar intake

SD_K_² was the interindividual variability of K intake

SD_Na_² was the interindividual variability of Na intake

**Selenium**

No EAR was set by Anses, thus a pseudo-EAR was calculated from the AI of 70 µg/d considering a standard variability of 15%. Thus the pseudo-EAR considered was 54 µg/d (15%). The upper bound value of 300µg/d was used in this study.

**Sodium**

Anses did not set any upper tolerable limit, but an upper bound value based on the median intake in the INCA2 population. In the population in this study, median sodium intakes were 3312 or 2483 mg/d with a 30% variability, and those values were chosen as reference values for men and women.

**Zinc**

As bioavailability of zinc was taken into account in this study, the requirement of 0.642 + 0.038 * b.w. mg/d was considered, as described in the Anses opinion. The upper bound limit of 25 mg/d was used in this study

.

**Table S1.** Odd-ratios (OR) [95% CI] and p-values^1^ of socio-demographic determinants of dietary patterns

|  | Processed meat eaters | | Poultry eaters | | Pork eaters | | Traditional eaters | | Milk drinkers | | Take-away eaters | | Beef eaters | | Fish eaters | |
| --- | --- | --- | --- | --- | --- | --- | --- | --- | --- | --- | --- | --- | --- | --- | --- | --- |
| Effect | **OR [CI 95%]** | **P-value** | **OR [CI 95%]** | **P-value** | **OR [CI 95%]** | **P-value** | **OR [CI 95%]** | **P-value** | **OR [CI 95%]** | **P-value** | **OR [CI 95%]** | **P-value** | **OR [CI 95%]** | **P-value** | **OR [CI 95%]** | **P-value** |
| Sex |  | 0.645 |  | **0.043** |  | 0.305 |  | 0.274 |  | **0.035** |  | 0.718 |  | **0.013** |  | 0.132 |
| M vs W | 0.9 [0.59 ; 1.39] | 0.645 | 1.67 [1.02 ; 2.75] | **0.043** | 1.22 [0.83 ; 1.8] | 0.305 | 0.84 [0.61 ; 1.15] | 0.274 | 0.65 [0.44 ; 0.97] | **0.035** | 0.92 [0.59 ; 1.44] | 0.718 | 1.59 [1.1 ; 2.29] | **0.013** | 0.64 [0.35 ; 1.15] | 0.132 |
| Age |  | 0.896 |  | 0.761 |  | 0.684 |  | **0.004** |  | 0.551 |  | **0.000** |  | 0.404 |  | 0.318 |
| 50+ vs 18-24 | 0.94 [0.45 ; 1.99] | 0.808 | 0.8 [0.31 ; 2.08] | 0.790 | 1.54 [0.63 ; 3.79] | 0.664 | 2.12 [1.04 ; 4.32] | **0.001** | 0.93 [0.44 ; 1.98] | 0.308 | 0.28 [0.14 ; 0.58] | **0.001** | 0.81 [0.4 ; 1.63] | 0.210 | 1.58 [0.52 ; 4.82] | 0.089 |
| 35-49 vs 18-24 | 0.92 [0.42 ; 2.01] | 0.917 | 0.94 [0.37 ; 2.41] | 0.597 | 1.73 [0.7 ; 4.26] | 0.256 | 1.5 [0.72 ; 3.1] | 0.414 | 1.25 [0.59 ; 2.63] | 0.437 | 0.4 [0.2 ; 0.78] | 0.060 | 0.96 [0.47 ; 1.95] | 0.831 | 1.02 [0.32 ; 3.25] | 0.863 |
| 24-34 vs 18-24 | 0.77 [0.33 ; 1.77] | 0.439 | 0.69 [0.25 ; 1.91] | 0.395 | 1.56 [0.61 ; 4] | 0.650 | 1.01 [0.47 ; 2.17] | 0.093 | 1.29 [0.6 ; 2.77] | 0.370 | 0.84 [0.43 ; 1.63] | 0.014 | 1.23 [0.59 ; 2.55] | 0.185 | 0.79 [0.23 ; 2.75] | 0.330 |
| Education |  | 0.157 |  | 0.428 |  | 0.106 |  | **0.005** |  | 0.162 |  | 0.393 |  | 0.796 |  | 0.684 |
| Post-secondary vs Primary | 0.57 [0.29 ; 1.1] | 0.261 | 0.9 [0.46 ; 1.76] | 0.689 | 0.55 [0.31 ; 1.01] | 0.164 | 1.83 [1.23 ; 2.73] | **0.048** | 1.28 [0.78 ; 2.12] | 0.865 | 0.9 [0.5 ; 1.6] | 0.746 | 0.86 [0.53 ; 1.42] | 0.733 | 0.76 [0.35 ; 1.66] | 0.392 |
| Secondary vs Primary | 0.68 [0.38 ; 1.22] | 0.751 | 0.63 [0.31 ; 1.27] | 0.229 | 0.69 [0.41 ; 1.17] | 0.792 | 1.6 [1.09 ; 2.36] | 0.349 | 1.52 [0.98 ; 2.35] | 0.159 | 0.67 [0.37 ; 1.19] | 0.223 | 0.88 [0.55 ; 1.41] | 0.819 | 1.1 [0.54 ; 2.21] | 0.503 |
| City size |  | 0.424 |  | 0.563 |  | 0.972 |  | 0.438 |  | 0.341 |  | 0.132 |  | 0.938 |  | 0.701 |
| rural vs > 100 000 | 1.41 [0.73 ; 2.72] | 0.210 | 0.73 [0.34 ; 1.59] | 0.322 | 1.07 [0.61 ; 1.89] | 0.836 | 1.33 [0.82 ; 2.15] | 0.199 | 1.1 [0.62 ; 1.98] | 0.854 | 0.51 [0.25 ; 1.05] | 0.156 | 0.9 [0.51 ; 1.6] | 0.731 | 0.68 [0.28 ; 1.66] | 0.426 |
| > 2000 vs > 100 000 | 0.93 [0.58 ; 1.5] | 0.287 | 1.09 [0.64 ; 1.85] | 0.352 | 1.03 [0.66 ; 1.61] | 0.989 | 1 [0.7 ; 1.43] | 0.414 | 1.34 [0.89 ; 2.02] | 0.199 | 0.69 [0.42 ; 1.13] | 0.879 | 0.97 [0.65 ; 1.46] | 0.898 | 0.9 [0.48 ; 1.68] | 0.775 |
| Loc. of the household |  | 0.075 |  | 0.592 |  | 0.283 |  | 0.671 |  | 0.895 |  | 0.057 |  | 0.987 |  | 0.088 |
| City center vs dispersed housing | 1.63 [0.64 ; 4.12] | 0.843 | 1.54 [0.57 ; 4.18] | 0.606 | 0.56 [0.3 ; 1.07] | 0.295 | 1.02 [0.57 ; 1.83] | 0.780 | 0.82 [0.43 ; 1.56] | 0.726 | 0.81 [0.39 ; 1.7] | 0.307 | 1.14 [0.56 ; 2.29] | 0.782 | 3.61 [0.78 ; 16.67] | 0.173 |
| Suburbs vs dispersed housing | 2.64 [1.1 ; 6.34] | **0.021** | 1.31 [0.49 ; 3.51] | 0.823 | 0.58 [0.32 ; 1.07] | 0.346 | 1.25 [0.71 ; 2.17] | 0.241 | 0.86 [0.46 ; 1.6] | 0.954 | 0.47 [0.22 ; 1.02] | 0.079 | 1.1 [0.56 ; 2.18] | 0.909 | 2.26 [0.49 ; 10.51] | 0.779 |
| Village vs dispersed housing | 1.95 [0.87 ; 4.35] | 0.501 | 1.76 [0.7 ; 4.44] | 0.262 | 0.65 [0.38 ; 1.12] | 0.809 | 1.01 [0.61 ; 1.68] | 0.712 | 0.8 [0.45 ; 1.44] | 0.665 | 0.53 [0.25 ; 1.1] | 0.282 | 1.1 [0.58 ; 2.07] | 0.926 | 4.42 [1.01 ; 19.24] | **0.035** |
| Occupational status |  | 0.424 |  | 0.563 |  | 0.972 |  | 0.438 |  | 0.341 |  | 0.132 |  | 0.938 |  | 0.701 |
| Low vs very high | 0.74 [0.41 ; 1.35] | 0.637 | 1.92 [0.87 ; 4.22] | 0.490 | 1.38 [0.78 ; 2.44] | 0.428 | 0.84 [0.5 ; 1.43] | 0.377 | 0.8 [0.43 ; 1.48] | 0.855 | 2.12 [1.04 ; 4.31] | 0.073 | 0.65 [0.36 ; 1.16] | 0.169 | 0.56 [0.19 ; 1.63] | 0.152 |
| Medium vs very high | 0.53 [0.32 ; 0.86] | 0.171 | 1.7 [0.84 ; 3.45] | 0.828 | 0.99 [0.59 ; 1.64] | 0.215 | 1.09 [0.74 ; 1.61] | 0.392 | 0.88 [0.55 ; 1.38] | 0.708 | 1.79 [0.98 ; 3.29] | 0.187 | 0.91 [0.57 ; 1.44] | 0.616 | 1.01 [0.52 ; 1.97] | 0.843 |
| High vs very high | 0.52 [0.22 ; 1.23] | 0.404 | 2.18 [0.88 ; 5.36] | 0.298 | 1.49 [0.73 ; 3.05] | 0.343 | 1.03 [0.62 ; 1.73] | 0.796 | 0.68 [0.34 ; 1.35] | 0.387 | 1.11 [0.44 ; 2.8] | 0.396 | 0.87 [0.45 ; 1.67] | 0.891 | 1.54 [0.6 ; 3.98] | 0.148 |

^1^ OR and p-values from logistic regression comparing the dietary pattern with the overall population, each variable independently of the others.

**Table S2.** Mean (± SD) probabilities of adequacy for components of the PANDiet score and ProtDiv-S

| **Nutrient** | **Processed meat eaters** | **Poultry eaters** | **Pork eaters** | **Traditionnal eaters** | **Milk drinkers** | **Take-away eaters** | **Beef eaters** | **Fish eaters** | **ProbF** |
| --- | --- | --- | --- | --- | --- | --- | --- | --- | --- |
| **PANDiet** | 57.99 ± 5.61 | 55.97 ± 6.41 | 56.47 ± 4.8 *- | 58.03 ± 5.76 | 58.88 ± 5.44 *+ | 53.71 ± 5.57 *- | 57.88 ± 6.61 | 61.79 ± 6.86 *+ | <.0001 |
| **AS** | 64.18 ± 11.22 | 62.39 ± 12.14 | 64.39 ± 9.11 | 65.92 ± 10.18 *+ | 67.32 ± 9.66 *+ | 58.35 ± 11.54 *- | 62.69 ± 11.66 | 67.97 ± 9.95 *+ | <.0001 |
| **Protein** | 0.98 ± 0.05 | 0.99 ± 0.03 | 0.99 ± 0.04 | 0.98 ± 0.07 | 0.98 ± 0.07 | 0.97 ± 0.08 *- | 0.98 ± 0.06 | 0.98 ± 0.06 | 0.0593 |
| **Total fat** | 0.8 ± 0.21 | 0.82 ± 0.25 | 0.86 ± 0.16 *+ | 0.86 ± 0.19 | 0.85 ± 0.19 | 0.86 ± 0.19 | 0.8 ± 0.25 *- | 0.8 ± 0.21 | 0.0040 |
| **LA** | 0.6 ± 0.34 | 0.67 ± 0.33 *+ | 0.56 ± 0.33 | 0.6 ± 0.32 | 0.54 ± 0.35 *- | 0.52 ± 0.36 | 0.56 ± 0.38 | 0.7 ± 0.31 | <.0001 |
| **ALA** | 0.08 ± 0.17 | 0.08 ± 0.18 | 0.08 ± 0.16 | 0.13 ± 0.23 | 0.08 ± 0.17 | 0.06 ± 0.12 *- | 0.07 ± 0.19 | 0.2 ± 0.26 *+ | 0.0002 |
| **DHA** | 0.32 ± 0.34 | 0.28 ± 0.33 | 0.31 ± 0.3 | 0.45 ± 0.34 *+ | 0.26 ± 0.32 *- | 0.22 ± 0.32 *- | 0.28 ± 0.36 | 0.57 ± 0.3 *+ | <.0001 |
| **EPA+DHA** | 0.27 ± 0.32 | 0.22 ± 0.31 | 0.26 ± 0.29 | 0.4 ± 0.34 *+ | 0.22 ± 0.3 | 0.19 ± 0.3 *- | 0.24 ± 0.34 | 0.49 ± 0.3 *+ | <.0001 |
| **Fibers** | 0.32 ± 0.34 | 0.22 ± 0.29 | 0.22 ± 0.25 *- | 0.26 ± 0.3 *+ | 0.2 ± 0.26 | 0.13 ± 0.21 *- | 0.19 ± 0.28 | 0.39 ± 0.34 *+ | <.0001 |
| **Vit A** | 0.69 ± 0.32 | 0.56 ± 0.37 | 0.66 ± 0.29 | 0.72 ± 0.28 *+ | 0.71 ± 0.3 | 0.54 ± 0.36 *- | 0.53 ± 0.37 *- | 0.7 ± 0.31 | <.0001 |
| **Vit B1** | 0.98 ± 0.04 | 0.97 ± 0.06 | 0.99 ± 0.02 *+ | 0.98 ± 0.05 | 0.99 ± 0.02 *+ | 0.97 ± 0.07 *- | 0.97 ± 0.07 *- | 0.97 ± 0.06 | <.0001 |
| **Vit B2** | 0.93 ± 0.11 | 0.85 ± 0.24 | 0.91 ± 0.15 | 0.91 ± 0.17 | 0.99 ± 0.04 *+ | 0.84 ± 0.23 *- | 0.88 ± 0.22 | 0.87 ± 0.22 | <.0001 |
| **Vit B3** | 1 ± 0 | 1 ± 0 | 1 ± 0 | 1 ± 0 | 1 ± 0 | 1 ± 0 | 1 ± 0 | 1 ± 0 | 0.7727 |
| **Vit B5** | 0.9 ± 0.16 | 0.9 ± 0.16 | 0.91 ± 0.12 | 0.92 ± 0.11 | 0.96 ± 0.07 *+ | 0.83 ± 0.2 *- | 0.91 ± 0.13 | 0.9 ± 0.15 | <.0001 |
| **Vit B6** | 0.71 ± 0.32 | 0.74 ± 0.31 *+ | 0.72 ± 0.29 | 0.68 ± 0.3 | 0.72 ± 0.3 | 0.5 ± 0.36 *- | 0.71 ± 0.33 | 0.71 ± 0.32 | <.0001 |
| **Vit B9** | 0.65 ± 0.34 | 0.59 ± 0.38 | 0.64 ± 0.29 | 0.73 ± 0.28 *+ | 0.7 ± 0.32 *+ | 0.53 ± 0.33 *- | 0.57 ± 0.36 *- | 0.72 ± 0.3 *+ | <.0001 |
| **Vit B12** | 0.77 ± 0.28 | 0.74 ± 0.31 *- | 0.74 ± 0.28 *- | 0.75 ± 0.28 | 0.85 ± 0.22 *+ | 0.73 ± 0.32 *- | 0.82 ± 0.25 *+ | 0.68 ± 0.33 | <.0001 |
| **Vit C** | 0.36 ± 0.4 | 0.36 ± 0.42 | 0.34 ± 0.34 | 0.38 ± 0.36 | 0.42 ± 0.38 *+ | 0.27 ± 0.38 *- | 0.32 ± 0.39 | 0.47 ± 0.37 *+ | <.0001 |
| **Vit D** | 0.02 ± 0.06 | 0.03 ± 0.11 | 0.02 ± 0.07 | 0.04 ± 0.08 *+ | 0.03 ± 0.08 | 0.02 ± 0.06 *- | 0.03 ± 0.1 | 0.03 ± 0.07 | 0.1019 |
| **Vit E** | 0.86 ± 0.19 | 0.89 ± 0.15 | 0.87 ± 0.17 | 0.91 ± 0.14 *+ | 0.9 ± 0.15 | 0.85 ± 0.19 *- | 0.83 ± 0.22 *- | 0.95 ± 0.1 *+ | <.0001 |
| **Calcium** | 0.52 ± 0.38 *- | 0.5 ± 0.41 *- | 0.59 ± 0.35 *- | 0.66 ± 0.33 *+ | 0.87 ± 0.21 *+ | 0.58 ± 0.35 *- | 0.5 ± 0.37 *- | 0.62 ± 0.35 | <.0001 |
| **Copper** | 0.86 ± 0.21 | 0.8 ± 0.3 | 0.88 ± 0.16 | 0.91 ± 0.15 *+ | 0.84 ± 0.22 | 0.82 ± 0.25 *- | 0.83 ± 0.25 | 0.91 ± 0.16 *+ | <.0001 |
| **Iodine** | 0.55 ± 0.3 *- | 0.52 ± 0.35 *- | 0.59 ± 0.29 | 0.62 ± 0.29 | 0.8 ± 0.22 *+ | 0.56 ± 0.31 *- | 0.56 ± 0.34 *- | 0.67 ± 0.26 *+ | <.0001 |
| **Iron** | 0.75 ± 0.25 *+ | 0.7 ± 0.29 | 0.72 ± 0.23 | 0.59 ± 0.27 *- | 0.64 ± 0.25 | 0.56 ± 0.31 *- | 0.76 ± 0.27 *+ | 0.62 ± 0.23 | <.0001 |
| **Magnesium** | 0.37 ± 0.35 | 0.37 ± 0.38 | 0.37 ± 0.3 | 0.42 ± 0.34 | 0.4 ± 0.31 | 0.32 ± 0.35 *- | 0.37 ± 0.35 | 0.48 ± 0.35 *+ | 0.0007 |
| **Manganese** | 0.88 ± 0.15 | 0.83 ± 0.18 | 0.87 ± 0.15 | 0.92 ± 0.12 *+ | 0.84 ± 0.17 *- | 0.84 ± 0.18 *- | 0.85 ± 0.18 | 0.92 ± 0.11 *+ | <.0001 |
| **Phosphorus** | 1 ± 0 | 1 ± 0 | 1 ± 0.02 | 1 ± 0.01 | 1 ± 0 | 1 ± 0.01 | 1 ± 0.04 | 1 ± 0 | 0.6386 |
| **Potassium** | 0.26 ± 0.25 *- | 0.31 ± 0.28 *+ | 0.32 ± 0.26 | 0.27 ± 0.26 *- | 0.34 ± 0.24 *+ | 0.2 ± 0.21 *- | 0.33 ± 0.29 | 0.44 ± 0.26 *+ | <.0001 |
| **Selenium** | 0.84 ± 0.17 | 0.84 ± 0.19 | 0.87 ± 0.12 | 0.86 ± 0.13 | 0.86 ± 0.13 | 0.8 ± 0.17 *- | 0.84 ± 0.16 | 0.88 ± 0.12 *+ | <.0001 |
| **Zinc** | 0.36 ± 0.26 | 0.35 ± 0.29 | 0.39 ± 0.27 | 0.29 ± 0.25 *- | 0.42 ± 0.27 | 0.35 ± 0.3 | 0.48 ± 0.28 *+ | 0.25 ± 0.24 *- | <.0001 |
| **MS** | 51.79 ± 14.07 | 49.56 ± 12.75 | 48.56 ± 10.95 | 50.14 ± 12.68 | 50.43 ± 12.39 | 49.07 ± 11.73 | 53.08 ± 14.35 *+ | 55.6 ± 12.78 *+ | 0.0013 |
| **Protein** | 0.97 ± 0.1 | 0.94 ± 0.17 *- | 0.96 ± 0.08 | 0.97 ± 0.09 | 0.98 ± 0.05 | 0.97 ± 0.11 | 0.97 ± 0.07 | 0.97 ± 0.08 | 0.0006 |
| **Total fat** | 0.66 ± 0.24 | 0.63 ± 0.26 | 0.58 ± 0.24 *- | 0.58 ± 0.25 *- | 0.62 ± 0.23 *+ | 0.61 ± 0.24 | 0.65 ± 0.27 | 0.66 ± 0.23 | <.0001 |
| **SFA** | 0.26 ± 0.28 *+ | 0.26 ± 0.31 | 0.15 ± 0.19 *- | 0.18 ± 0.23 | 0.13 ± 0.2 *- | 0.15 ± 0.19 | 0.23 ± 0.29 | 0.34 ± 0.3 *+ | <.0001 |
| **Cholesterol** | 0.41 ± 0.33 | 0.33 ± 0.32 *- | 0.38 ± 0.3 | 0.48 ± 0.33 | 0.48 ± 0.32 | 0.48 ± 0.32 | 0.45 ± 0.37 | 0.57 ± 0.3 *+ | 0.0002 |
| **Sugar** | 0.74 ± 0.32 | 0.66 ± 0.39 | 0.7 ± 0.31 | 0.72 ± 0.31 | 0.69 ± 0.34 | 0.61 ± 0.39 *- | 0.73 ± 0.34 | 0.65 ± 0.31 | 0.0275 |
| **Sodium** | 0.1 ± 0.15 | 0.16 ± 0.2 | 0.14 ± 0.18 | 0.1 ± 0.15 *- | 0.12 ± 0.15 | 0.12 ± 0.16 | 0.16 ± 0.21 *+ | 0.15 ± 0.16 *+ | <.0001 |
| **ProtDiv-S** | 69.06 ± 12.51 | 68.19 ± 14.08 | 68.03 ± 12.33 | 70.13 ± 12.01 *+ | 67.58 ± 12.89 | 63.72 ± 13.2 *- | 63.88 ± 14.87 *- | 71.41 ± 11.88 *+ | <.0001 |

*+/*-, significantly higher or lower than the overall population mean; ProbF, p-value of the ANOVA testing the dietary pattern effect on the probability of adequacy

.

**Table S3.** Mean (± SD) contribution of food groups^1^ to protein intake

| **Nutrient** | **Processed meat eaters** | **Poultry eaters** | **Pork eaters** | **Traditional eaters** | **Milk drinkers** | **Take-away eaters** | **Beef eaters** | **Fish eaters** | **ProbF** |
| --- | --- | --- | --- | --- | --- | --- | --- | --- | --- |
| ***Animal*** | 67.79 ± 7.74 | 72.79 ± 7.94 *+ | 71.03 ± 7.05 *+ | 66.22 ± 7.87 *- | 71.03 ± 5.63 *+ | 66.36 ± 8.03 *- | 69.64 ± 8.04 | 65.37 ± 10.52 *- | <.0001 |
| Meat (except poultry) | 18.75 ± 9.06 | 14.13 ± 7.8 *- | 24.91 ± 8.74 *+ | 11 ± 6.3 *- | 14.98 ± 8.67 *- | 16.91 ± 10.18 | 27.46 ± 9.01 *+ | 13.68 ± 8.63 *- | <.0001 |
| Poultry | 11.05 ± 8.39 *+ | 26.74 ± 9.96 *+ | 7.6 ± 7.29 *- | 8.52 ± 6.14 | 8.33 ± 5.83 *- | 5.99 ± 5.96 *- | 6.31 ± 7.09 *- | 9.65 ± 6.46 | <.0001 |
| Game | 0.17 ± 1.18 | 0.07 ± 0.82 | 0.17 ± 0.84 | 0.44 ± 2.04 | 0.14 ± 1.2 | 0.16 ± 1.48 | 0.4 ± 2.68 | 0.2 ± 1.13 | 0.6157 |
| Offal | 2.73 ± 4 *+ | 0.56 ± 1.98 | 0.71 ± 1.84 | 0.58 ± 1.48 | 0.69 ± 1.88 | 0.46 ± 2.23 | 0.35 ± 1.3 *- | 0.53 ± 1.93 | <.0001 |
| Delicatessen | 9.2 ± 6.06 | 7.91 ± 5.04 *- | 9.26 ± 5.18 *+ | 9.3 ± 5.81 *+ | 8.33 ± 4.74 | 8.9 ± 6.22 | 8.02 ± 6.14 | 5.65 ± 4.38 *- | <.0001 |
| Fish | 6.95 ± 6.32 | 5.22 ± 5.54 *- | 6.87 ± 6.08 | 9.58 ± 6.8 *+ | 5.77 ± 5.03 *- | 6.26 ± 6.08 | 6.03 ± 5.59 *- | 14.59 ± 8.47 *+ | <.0001 |
| Milk | 3.71 ± 3.8 *- | 3.62 ± 3.57 *- | 4.69 ± 4.07 *- | 3.65 ± 3.24 *- | 15.04 ± 5.7 *+ | 6.23 ± 5.3 | 4.89 ± 4.61 *- | 4.56 ± 2.98 *- | <.0001 |
| Yogurts | 3.3 ± 3.48 *- | 3.48 ± 4.13 | 3.67 ± 3.11 | 5.12 ± 4.92 *+ | 4.44 ± 3.73 | 2.7 ± 3.48 *- | 4.07 ± 4.64 | 6.16 ± 4.25 *+ | <.0001 |
| Cheese | 8.25 ± 6.17 *- | 7.92 ± 5.45 *- | 9.4 ± 5.5 *- | 13.27 ± 6.29 *+ | 8.61 ± 4.86 *- | 13.54 ± 6.6 *+ | 7.85 ± 5.06 *- | 6.72 ± 4.16 *- | <.0001 |
| Other dairy | 0.35 ± 0.31 *- | 0.36 ± 0.3 *- | 0.42 ± 0.3 | 0.48 ± 0.38 | 0.54 ± 0.37 *+ | 0.52 ± 0.5 *+ | 0.4 ± 0.35 | 0.47 ± 0.37 | <.0001 |
| Eggs | 3.32 ± 2.14 *- | 2.79 ± 2.15 *- | 3.33 ± 2.29 | 4.27 ± 2.91 *+ | 4.16 ± 2.68 | 4.68 ± 3.2 *+ | 3.86 ± 2.95 | 3.16 ± 1.92 *- | <.0001 |
| ***Plant*** | 32.21 ± 7.74 | 27.21 ± 7.94 *- | 28.97 ± 7.05 *- | 33.78 ± 7.87 *+ | 28.97 ± 5.63 *- | 33.64 ± 8.03 *+ | 30.36 ± 8.04 | 34.63 ± 10.52 *+ | <.0001 |
| Cereals | 21.29 ± 6.85 | 18.34 ± 6.93 *- | 19.49 ± 6.36 *- | 22.22 ± 6.71 *+ | 18.81 ± 5.08 *- | 23.15 ± 5.9 *+ | 20.98 ± 7.53 | 22 ± 8.26 | <.0001 |
| Potatoes | 1.86 ± 1.25 | 1.66 ± 1.16 *- | 1.85 ± 1.27 | 1.81 ± 1.18 | 1.79 ± 1.05 | 1.77 ± 1.24 | 1.99 ± 1.27 | 2.01 ± 1.31 | 0.0904 |
| Fruit | 1.97 ± 1.93 | 1.63 ± 1.4 | 1.58 ± 1.04 *- | 2.29 ± 2.13 *+ | 1.73 ± 1.14 | 2 ± 2.07 | 1.72 ± 1.6 | 2.41 ± 1.6 *+ | <.0001 |
| Vegetables | 2.83 ± 1.58 | 2.49 ± 1.55 | 2.69 ± 1.25 | 3.32 ± 1.52 *+ | 2.73 ± 1.31 | 2.38 ± 1.33 *- | 2.51 ± 1.41 *- | 3.7 ± 1.75 *+ | <.0001 |
| Nuts and seeds | 1.05 ± 1.89 | 0.51 ± 1.01 | 0.73 ± 1.2 | 1 ± 1.41 *+ | 0.72 ± 1 | 0.89 ± 1.36 | 0.67 ± 1.3 | 1.12 ± 1.3 | 0.0345 |
| Legumes | 1.52 ± 1.96 *+ | 1.11 ± 1.92 | 1.03 ± 1.51 | 1.07 ± 1.6 | 0.84 ± 1.49 | 0.95 ± 2.34 | 0.82 ± 1.73 *- | 1.21 ± 2.26 | 0.0302 |
| Other | 1.14 ± 0.87 *- | 1.03 ± 0.84 *- | 1.14 ± 0.69 *- | 1.44 ± 0.89 | 1.78 ± 1.26 *+ | 1.7 ± 1.28 *+ | 1.25 ± 0.9 *- | 1.39 ± 1.43 | <.0001 |
| Spices | 0.54 ± 0.47 | 0.45 ± 0.33 *- | 0.47 ± 0.35 | 0.62 ± 0.82 | 0.56 ± 0.51 | 0.79 ± 0.44 *+ | 0.44 ± 0.42 *- | 0.79 ± 0.79 *+ | <.0001 |

*+/*-, significantly higher or lower than the overall population mean; ProbF, p-value of the ANOVA testing the dietary effect on the probability of adequacy.

^1^ Food groups after breaking down composite foods into ingredients using recipes

**References**

1. Perignon, M.; Barré, T.; Gazan, R.; Amiot, M.-J.; Darmon, N. The bioavailability of iron, zinc, protein and vitamin a is highly variable in French individual diets: Impact on nutrient inadequacy assessment and relation with the animal-to-plant ratio of diets. *Food Chem.*, In press.

2. Gibson, R.S.; Bailey, K.B.; Gibbs, M.; Ferguson, E.L. A review of phytate, iron, zinc, and calcium concentrations in plant-based complementary foods used in low-income countries and implications for bioavailability. *Food Nutr. Bull.* **2010**, *31*, S134-146.

3. Sarwar Gilani, G.; Wu Xiao, C.; Cockell, K.A. Impact of antinutritional factors in food proteins on the digestibility of protein and the bioavailability of amino acids and on protein quality. *Br. J. Nutr.* **2012**, *108*.

4. EFSA Panel on Dietetic Products, Nutrition and Allergies (NDA),. Scientific opinion on dietary reference values for vitamin a. *EFSA Journal* **2015**, *13*, 4028.

5. Trumbo, P.; Yates, A.A.; Schlicker, S.; Poos, M. Dietary reference intakes: Vitamin a, vitamin k, arsenic, boron, chromium, copper, iodine, iron, manganese, molybdenum, nickel, silicon, vanadium, and zinc. *J. Am. Diet. Assoc.* **2001**, *101*, 294-301.

6. EFSA Panel on Dietetic Products, N.a.A.N. Scientific opinion on dietary reference values for calcium. *EFSA Journal* **2015**, *13*, 4101.

7. Miller, L.V.; Krebs, N.F.; Hambidge, K.M. A mathematical model of zinc absorption in humans as a function of dietary zinc and phytate. *J. Nutr.* **2007**, *137*, 135-141.

8. Armah, S.M.; Carriquiry, A.; Sullivan, D.; Cook, J.D.; Reddy, M.B. A complete diet-based algorithm for predicting nonheme iron absorption in adults. *J. Nutr.* **2013**, *143*, 1136-1140.

9. Hallberg, L.; Hulthen, L. Prediction of dietary iron absorption: An algorithm for calculating absorption and bioavailability of dietary iron. *Am. J. Clin. Nutr.* **2000**, *71*, 1147-1160.

10. FAO Expert Consultation. Dietary protein quality evaluation in human nutrition. *FAO Food Nutr. Pap.* **2011**, *92*.

11. Tome, D. Digestibility issues of vegetable versus animal proteins: Protein and amino acid requirements--functional aspects. *Food Nutr. Bull.* **2013**, *34*, 272-274.

12. Oberli, M.; Marsset-Baglieri, A.; Airinei, G.; Sante-Lhoutellier, V.; Khodorova, N.; Remond, D.; Foucault-Simonin, A.; Piedcoq, J.; Tome, D.; Fromentin, G.*, et al.* High true ileal digestibility but not postprandial utilization of nitrogen from bovine meat protein in humans is moderately decreased by high-temperature, long-duration cooking. *J. Nutr.* **2015**, *145*, 2221-2228.

13. Dietary Guidelines Advisory Committee. *Report of the dietary guidelines advisory committee on the dietary guidelines for americans, 2010, to the secretary of agriculture and the secretary of health and human services*; Washington DC, 2010.

14. EFSA Panel on Dietetic Products, Nutrition and Allergies (NDA),. Scientific opinion on dietary reference values for thiamin. *EFSA journal* **2016**, *14*, 4653.

15. EFSA Panel on Dietetic Products, Nutrition and Allergies (NDA),. Scientific opinion on dietary reference values for vitamin b6. *EFSA Journal* **2016**, *14*, e04485-n/a.

16. EFSA Panel on Dietetic Products, Nutrition and Allergies (NDA),. Scientific opinion on dietary references values for phosphorus. *EFSA journal* **2015**, *13*, 4185.
